# Review on the Polymeric and Chelate Gel Precursor for Li-Ion Battery Cathode Material Synthesis

**DOI:** 10.3390/gels10090586

**Published:** 2024-09-12

**Authors:** Mobinul Islam, Md. Shahriar Ahmed, Muhammad Faizan, Basit Ali, Md Murshed Bhuyan, Gazi A. K. M. Rafiqul Bari, Kyung-Wan Nam

**Affiliations:** 1 Department of Energy & Materials Engineering, Dongguk University, Pildong-ro 1-gil, Jung-gu, Seoul 04620, Republic of Koreafaizijaff@gmail.com (M.F.); basitalikhan077@gmail.com (B.A.); 2 School of Mechanical Smart and Industrial Engineering, Gachon University, Seongnamdaero, Sujeong-gu, Seongnam-si 13120, Gyeonggi-do, Republic of Koreagrafiqulbari@gachon.ac.kr (G.A.K.M.R.B.)

**Keywords:** gel precursor, sol-gel, chelating agent, metal-citrate gel, polymeric gel, lithium-ion battery, cathode material

## Abstract

The rapid design of advanced materials depends on synthesis parameters and design. A wide range of materials can be synthesized using precursor reactions based on chelated gel and organic polymeric gel pathways. The desire to develop high-performance lithium-ion rechargeable batteries has motivated decades of research on the synthesis of battery active material particles with precise control of composition, phase-purity, and morphology. Among the most common methods reported in the literature to prepare precursors for lithium-ion battery active materials, sol-gel is characterized by simplicity, homogeneous mixing, and tuning of the particle shape. The chelate gel and organic polymeric gel precursor-based sol-gel method is efficient to promote desirable reaction conditions. Both precursor routes are commonly used to synthesize lithium-ion battery cathode active materials from raw materials such as inorganic salts in aqueous solutions or organic solvents. The purpose of this review is to discuss synthesis procedure and summarize the progress that has been made in producing crystalline particles of tunable and complex morphologies by sol-gel synthesis that can be used as active materials for lithium-ion batteries.

## 1. Introduction

The concept of optimizing the synthesis of advanced inorganic materials is a grand challenge that can speed up the process of discovery. Many design variables interact with one another in synthesis, leading to complexity. For each element in the target material (oxides, hydroxides, carbides, etc.), these design variables include a variety of possible precursor candidates [1,2,3,4]. There are several other factors that contribute to the complexity of synthesis. These factors include the conditions used in the experiment (temperature, atmosphere, etc.) and the order in which operations are performed, i.e., mixing, firing, reducing, etc. A successful synthesis requires the careful selection of a combination of these experimental variables [5]. When preparing inorganic materials, such as metal oxides or carbides, it is typically a matter of mixing powder reactants and heating them to form the desired compound. This approach is well known as the solid-state method [6]. However, this method does have some drawbacks, even though reaction conditions are relatively easy to attain. The limited mass transport of powders leads to inhomogeneity of starting materials, making it difficult to achieve complete conversion in a mixture of two or more powders. If reactant diffusion is restricted, areas of unreacted starting materials will remain. The initial reaction therefore occurs at the edges of adjacent particles. By using ball-milling to decrease the particle size and increase the sample surface area, some of these problems can be overcome [7,8]. However, repeated sample milling steps or extended heating may be necessary. The most important issue is that solid-state methods often present difficulties in controlling particle morphology. The development of solution techniques has opened up a range of alternatives to solid-state chemistry, including coprecipitation, hydrothermal/solvothermal approaches, and the sol-gel method [9]. Sol-gel chemistry has some significant advantages. These include the ability to produce a solid-state material by mixing reactants at an atomic level, which allows for the successful synthesis of inorganic materials at a lower processing temperature and with a shorter process time frame [10,11]. In addition, particle size and morphology are expected to be more controllable with sol-gel chemistry. This is a synthesis process in which a liquid, called a “sol”, is transformed into a solid, called a “gel”, by the removal of the solvent from it to achieve a specific result. In terms of precursors for the sol-gel synthesis, the metallic alkoxides are the most used. In addition, gels precursor with some degree of order and structure have made some of the most interesting progress in the sol-gel field. The synthesis parameters and design play a big role in rapid design of advanced materials. Gel precursors can produce a wide range of materials. There are two common types of gel precursors—chelate gel and organic polymeric gel—that involve the use of small molecules (often chelating agents) and polymers to modify the hydrolysis chemistry of metal ions [12,13].

**Figure 1:**
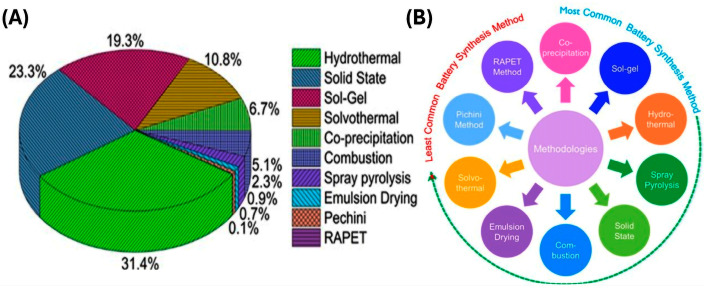
(**A**) The percentage of publications on the topic of lithium-ion batteries based on various synthesis techniques utilized in the literature. (**B**) Synthesis methods in order of their frequency of use, reproduced with permission from ref. [38]; copyright, Elsevier.

Lithium-ion batteries (LIBs) find extensive use across diverse fields, encompassing smartphones, laptops, various electronic equipment, electric vehicles (EVs), and grid storage systems worldwide [14,15]. There is no doubt that LIBs have advanced a lot since Sony introduced them to the world in the early 1990s [16]. Since then, there has been a tremendous improvement in their performance in terms of capacity, power density, cycle life, and safety. The continuous introduction of advanced materials has significantly contributed to this progress, which provides higher potential, enhanced volumetric and gravimetric capacity, and improved thermal safety [17]. LIBs have several components: an anode electrode (such as graphite), a cathode electrode (such as layered transition metal oxide) separated by a separator made up of a porous membrane (such as glass fiber, cellulose, or microporous polymer). The electrodes are immersed in non-aqueous or aprotic electrolytes consisting of lithium salts dissolved in an organic solvent [18,19]. It is undeniable that the cathode material is the most critical component of LIBs, as it plays a major role in both the electrochemical properties and the safety of the battery. In the advancement of LIBs in addition to making the right decision for a certain application, the choice of a cathode is vital. Over the past 30 years, numerous cathode materials, including lithium cobalt oxide (LiCoO_2_ or LCO), lithium iron phosphate (LiFePO_4_ or LFP), lithium manganese oxide (LiMn_2_O_4_ or LMO), lithium nickel cobalt aluminum oxide (NCA), and lithium nickel cobalt manganese oxide (NCM), have been developed and successfully introduced into the commercial market [20,21,22]. The preparation of these cathode materials has been accomplished using various techniques of wet chemical synthesis and solid-state synthesis. These methods encompass the sol-gel approach, hydrothermal/solvothermal synthesis, spray pyrolysis, solid-state reaction, emulsion drying, combustion, coprecipitation, and template method [23,24,25,26,27,28,29,30,31,32,33,34,35,36,37]. In Figure 1, the methods are arranged based on their prevalence, with the sol-gel approach being one of the most common methods besides solid-state and hydrothermal methods, and others following in decreasing order of frequency, as indicated by the literature surveyed [38]. Despite solid-state chemistry and hydrothermal chemistry being among the most widely used synthesis techniques in the literature related to Li-ion batteries in the past, sol-gel chemistry has grown in popularity over the last 10–15 years. Sol-gel synthesis is an attractive method for producing Li-ion battery materials due to its ability to create nanostructures with controlled porosity, surface area, and composition. Furthermore, it is relatively inexpensive. This review aims to provide a concise overview of published sol-gel synthesis papers that produce some preferential LIBs’ cathode active materials.

## 2. Gels Formation via Hydrolysis and Condensation

### 2.1. Basic Procedure

**Scheme 1:**
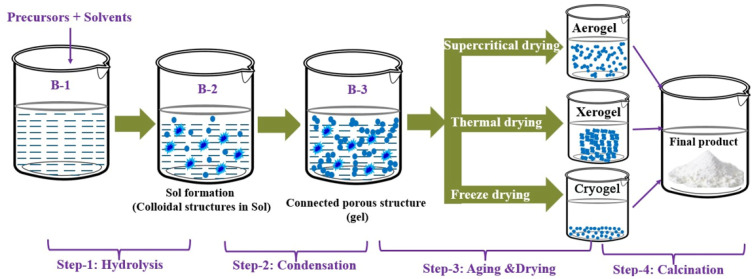
Schematic representation of different sol–gel processes from precursor to gel formation.

The hydrolysis and condensation of metal alkoxide are two steps that occur in the ‘traditional’ sol–gel chemistry process [39]. The ‘sol’, which is often broadly described as a colloidal suspension, is something that is formed by the hydrolysis reaction during the first step of the process. In the second step, the ‘gel’ state formed, which can be described simply as a three-dimensional non-fluid network extending through a fluid medium [40,41,42]. The first sol–gel reaction was observed in the 19th century when an alkoxide compound made from SiCl_4_ was exposed to air and started to form a gel [43]. The moisture in the atmosphere causes the silicon alkoxide to first hydrolyze and then condense. Using acid or base catalysis, this whole process can be carefully tuned to produce gels with a wide range of structural properties. When acidic conditions prevail, hydrolysis steps become progressively slower, and when basic conditions prevail, the process becomes faster [44,45]. A similar process can also be followed in sol–gel chemistry for other metal alkoxides. Water reacts violently with many transitions metal alkoxides due to the higher rate of hydrolysis, making most require extra care and handling. For example, when titanium alkoxides are exposed to water, they undergo a vigorous reaction that results in the formation of ill-defined titanium–oxo/hydroxo precipitates instead of the desired metal alkoxide precursor [46]. To slow down the hydrolysis of titanium alkoxides, additives are required; this contrasts with silicon alkoxides, for which catalysts are introduced to enhance condensation and hydrolysis. Due to the relative rates of the hydrolysis and condensation reactions, the resultant gel structure varies significantly depending on the concentration of catalyst, the ratio between alkoxide and water, and the electron-donating or electron-withdrawing ability of the R-group (alkyl/aryl chain) [47]. There are also a couple of physical factors that can affect alkoxide suitability for sol-gel chemistry, such as volatility and viscosity [48]. Lithium alkoxides are usually regarded as ionic compounds. When moist air exposes lithium alkoxide, it undergoes stepwise hydrolysis. During the hydrolysis process, the alkoxide anion stays intact due to its very low Brønsted basicity and does not deprotonate the water molecules easily [49]. It is, however, simple for lithium alkoxide to form double metal alkoxide in alcohol [50]. This hydrolysis process is important in the production of lithium compounds, such as LiNbO_3_ [51]. Lithium alkoxide is also widely used in the preparation of lithium-containing polymers. Rechargeable lithium-ion battery cells can benefit from adding lithium alkoxide as an electrolyte additive [52]. Another critical step is to dry the gel, i.e., to turn the solvent-filled gel into a solid. Solvent can be evaporated quite simply; however, capillary forces are applied to the gel when the liquid flows through it, causing it to collapse. Gels can be aged for a long time before drying to mitigate this problem to some extent. As a result of this uncontrolled drying process, xerogels are created. Xerogels have a large surface area due to their many small pores, but if structure-directing agents are not added, the porosity is generally disordered [53,54]. An aerogel can be produced by drying gels under supercritical conditions if a larger pore volume is needed. In an aerogel, up to 98% of the pockets are filled with air (or another gas) [55,56]. Additionally, freeze drying can form cryogel, a gel with porosity between xerogel and aerogel [57]. Scheme 1 represents the transition from the initial solution mixture to different gel formations based on various drying conditions. In the end, heat treatment is the step that is necessary to form the final product and is accomplished by densifying the material to produce a ceramic monolith or by converting it into crystalline form.

There is no question that this ‘traditional’ sol–gel chemistry is one of the most extensively studied and used fields of materials chemistry today [58]. Despite this, there is a significant limitation associated with alkoxide-based sol–gel chemistry. There are many elements that do not readily form alkoxides, thus restricting the above hydrolysis and condensation chemistry [59]. Furthermore, many of these compounds are highly reactive, which limits their application. With careful handling, some metal alkoxides sensitive to moisture can be used in sol–gel synthesis, but they may not work with water-soluble templates and structure-directing agents. The use of aqueous metal salts instead of alkoxides has been developed as an alternative method. Although the chemistry of these alternative approaches is quite different, their ultimate goal remains the same: the controlled transformation of the solution-phase precursors into metal oxide or other ceramic structures. These alternatives approaches can be broadly categorized as two common types of sol–gels: chelate gel and organic polymeric gel, based on their synthesis mechanisms [60]. 

### 2.2. Small Molecule Gelators

The incorporation of small organic molecules as chelating agents plays a crucial role in modulating the hydrolysis of metal ions during sol–gel synthesis [61]. In aqueous solutions, water molecules coordinate with transition metal ions via bonding orbitals. This coordination facilitates the transfer of electron density to the metal ions vacant d-orbitals, resulting in the weakening of the O-H bonds in the bound water molecules. This bond weakening can lead to deprotonation or hydrolysis, depending on the solution’s pH level (Equation (1)).

[M(OH_2_)]*^z^*^+^ ⇔ [M−OH]^(*z*−1)+^ + H^+^ ⇔ [M=O]^(*z*−2)+^ + 2H^+^ (1)

For highly reactive metals, controlling water content and using nonaqueous solvents are essential strategies to mitigate the rapid rate of hydrolysis [39,40]. In contrast, with less reactive metals, forming an aqueous solution is more straightforward, and pH levels can be effectively managed to control hydrolysis. Acidic conditions (pH < 7) tend to promote the formation of hydroxo ligands or even prevent hydrolysis entirely. This is because acidic conditions reduce the solubility of hydroxide ions, which are required for the hydrolysis process. The basic conditions (pH > 7) favor the formation of oxo ligands in principle because they shift equilibrium to the right [62]. Additionally, highly charged metal cations facilitate hydrolysis by weakening the O-H bonds in coordinated water molecules [39]. 

The synthesis of most metal oxides and ceramics typically involves thermal treatment. In general, the consequence of simply drying a metal salt solution is either the precipitation of the original salt from the solution or the formation of amorphous oxides or hydroxides. The precipitation process typically involves solvent evaporation, resulting in a dry solid. Alternatively, the metal salt solution can be heated to high temperatures, which causes the oxides or hydroxides to decompose, forming undesired solid particles. These occurrences have been prevented using small molecules to form stable metal complexes in water or organic solvents that resemble gels. As an example, ethylenediaminetetraacetic acid (EDTA) is commonly used as a chelating agent. The main function of such small molecules used in sol–gel chemistry is to change the hydrolysis equilibrium of dissolved metals [61]. For instance, when EDTA is added to aqueous iron, the equilibrium constant of hydrolysis is significantly reduced, making hydrolysis considerably less favorable. Consequently, when the solvent from metal–chelate solutions is removed, homogeneous glassy solids or resins (‘gel’-like precursor) are produced rather than precipitates [63,64]. Metal oxides, metal nitrides, and metal carbides can be formed as powders or nanostructures from these gel precursors by heating [65,66,67]. 

Citric acid is the most used small organic molecule to create chelated gel precursors for active cathode materials in Li-ion batteries through sol–gel chemistry [68,69,70]. This weak triprotic acid contains three dissociable carboxylic acid groups. In the typical procedure, citric acid and metal salts (such as nitrates) dissolve in an aqueous solution, which then forms a viscous solution or gel after the solvent evaporates through gentle heating. The resulting gel is then subjected to a thermal treatment, commonly known as calcination, at moderate temperatures. This process decomposes the organic components and crystallizes the metal oxides. The final product is a fine, homogeneous powder with the desired properties. To avoid the precipitation of individual metal hydroxides, the pH must be adjusted to facilitate the formation of stable metal citrate complexes. Citrate cations are more likely to bind to ions when the pH is modified by bases like ammonia or ethylene diamine. This modification increases the solubility of the citrate compound, allowing it to be more easily dissolved in water. It also increases citrate’s affinity for specific cations. This makes citrate an invaluable compound in the gel precursor-based synthesis method [70,71]. The metal nitrate precursors play a crucial role in the citrate sol–gel method. When heated, these nitrate counterions promote a fast, self-propagating combustion that starts at about 200 °C. The nitrate acts as an oxidant, and the citrate serves as the fuel [72]. There is a relationship between the pH of the precursor solution and the crystallinity and morphology of the resulting powders. When pH levels rise, oxide products may have more open networks and porousness [73,74]. This network structure allows for better Li^+^ ion diffusion, which leads to improved electrochemical kinetics. The increased porosity also allows for a larger surface area, which can be beneficial for electrolyte infiltration in battery applications. During the combustion reaction of nitrate with organic compounds, a large quantity of gases is generated, which often results in ‘sponge-like’ product. A build-up of NH_4_NO_3_ occurs if pH is modified by adding ammonia, which helps speed up combustion and decomposition of NH_4_NO_3_ into NOx and O_2_, making the product more porous [74,75]. Other mono- and di-carboxylic acids, such as tartaric acid, glycolic acid, and oxalic acid, are also utilized as gelators in similar contexts [76]. These acids form hydrogen bonds with water molecules, forming a rigid network. During this process, metal cations are trapped and immobilized in this network, resulting in a gel precursor.

To transform the metal–citrate ‘gel’ into metal oxide, a simple pyrolysis in air is necessary. During this process, the organic component combusts at around 200–400 °C, depending on the metal counterion and any additives present. When nucleation occurs during the first stages of synthesis, the presence of the organic matrix ensures a small crystallite size by distributing the sites evenly and in large numbers. This matrix is also essential in ternary or quaternary systems so that the metals stay mixed atomically [77]. However, using an inert atmosphere can yield ceramic or carbon composites, with citrate providing the carbon source. The synthesis of carbon/LiFePO_4_ serves as an example, where the reducing conditions maintain the ferrous state of the iron precursor throughout the synthesis [78]. 

Another commonly used chelator and ‘fuel’ is glycine, which has a lower ignition temperature than citrate [79,80]. ‘Fluffy’ powders are formed by burning glycine and nitrate mixtures. In some cases, however, the reaction can be explosive due to its high exothermic nature. This issue can be mitigated by modifying the glycine–nitrate method. In a glycine–nitrate mixture, the cellulose fibers act as a microreactor to ensure the particles remain very small and help to alleviate the occurrence of violent reactions and gas release [81]. Additionally, glucose and amino acids such as glutamine and histidine can be used as small molecule gelators in sol–gel synthesis [82,83]. It is important to note that some chelating agents, such as EDTA, have a higher decomposition temperature than citric acid [84]. In contrast to the other examples, the epoxy ‘sol–gel’ method involves slightly different chemistry. Nevertheless, the overall approach still entails the addition of a small molecule (propylene oxide) to affect the hydrolysis of a dissolved metal salt. The role of propylene oxide in this approach differs from that of chelating agents, as it acts as a proton scavenger, which promotes metal–oxo bond formation. Similarly, there have also been instances where urea has been used during standard fuel/nitrate sol–gel combustion synthesis in order to form metal oxides as well.

### 2.3. Polymeric Gelators

In situ polymerization around metal ions is highly effective for achieving a homogeneous mixture and stabilization of metals within a covalent framework or “gel”. One notable technique, the Pechini method [85], modifies the traditional sol–gel process by incorporating ethylene glycol into a solution of metal and citrate complexes. This method induces poly-esterification between citrate and ethylene glycol, forming a covalent polymer network that traps the metal ions. This method was devised with the intention of delaying the thermal decomposition of the organic matrix, which would allow more control over the ceramic product during its growth. In the Pechini method, two or more metals may be dispersed uniformly throughout a polymeric precursor. This is a particularly significant advantage [86]. Using this process, citric acid can also be substituted for other di-, tri-, or tetra-carboxylic acids, and EDTA and/or ethylene glycol can be substituted for other polyols to increase the range of materials that can be used [77,87]. Many synthetic and natural polymers also exhibit strong interactions with metal ions. These interactions are crucial for the sol–gel synthesis process as they can influence the structure and properties of the resulting materials. By binding with metal ions, polymers can help control the gelation rate and the morphology of the formed gel. This can lead to enhanced mechanical strength and stability in the final product. Suitable polymers for sol–gel processes must dissolve in at least one solvent—ideally water—and possess functional groups capable of binding metal ions. Using some polymers that form ordered superstructures may allow ceramics to have controlled morphologies. Many sol–gel synthesis studies use synthetic polymers such as polyvinyl alcohol (PVA). A typical approach involves mixing aqueous metal salts (such as nitrates) with PVA to create a homogeneous precursor, which is then heated at moderate temperatures (approximately 80 °C) to form a gel. A metal oxide ceramic is typically produced by drying this gel, then heating it in a furnace [88]. Polymers facilitate the control of particle size (~25 nm). The sol–gel synthesis of ceramics has also used several other synthetic polymers, such as polyethylene glycol (PEG) or polyvinylpyrrolidone (PVP), in addition to PVA [89,90]. The natural polymers usually used in sol–gel chemistry have focused on polysaccharides and polypeptides such as starch, dextran, chitin/chitosan, alginate, and gelatin [77].

## 3. Sol–Gel-Derived Cathodes and Associated Electrochemical Performance

In the past few decades, there has been significant interest in using the sol–gel method to synthesize metal oxides and phosphates, as well as many other materials, including diverse cathode materials of LIBs with large surface areas [91,92,93]. It is possible to insert even a small number of dopants into preferred locations using the sol–gel process due to the molecular-level mixing of dispersed ions [94]. There are several common chelating agents that have been used in the preparation of LIB cathode materials, including ethylene glycol, ascorbic acid, and citric acid. These chelating agents also contribute to in situ carbon generation [91]. The sol–gel approach for the synthesis of several state-of-art LIBs cathodes such as LiCoO_2_, LiNiO_2_, and LiMn_2_O_4_ has already been comprehensively reviewed by Liu et al. [95]. There are various types of sol–gel precursor establishments, and we are going to attempt to classify many of them in this review for preparing three commercially viable LIB cathode materials, such as NCM, NCA, and LiFePO_4_. LIBs performance is assessed by their charge/discharge capacities. Battery C-rates determine how quickly a battery charges and discharges. The capacity of a battery is commonly rated at 1 C, meaning that a fully charged battery rated at 1 Ah (ampere-hour) should provide 1 A for one hour. The same battery discharging at 0.5 C should provide 500 mA for two hours, and at 2 C, it delivers 2 A for 30 min.

### 3.1. Lithium Nickel Cobalt Manganese Oxide (NCM)

LiNi_1/3_Co_1/3_ Mn_1/3_O_2_ (abbreviated NCM111) stands out as one of the extensively studied layered materials, capable of delivering a specific capacity of 160 mAh g^−1^ when charged to 4.3 V [96]. This cathode material is currently integrated into various commercial Li-ion batteries, serving as a partial replacement for the very first commercial cathode, LiCoO_2_. 

**Figure 2:**
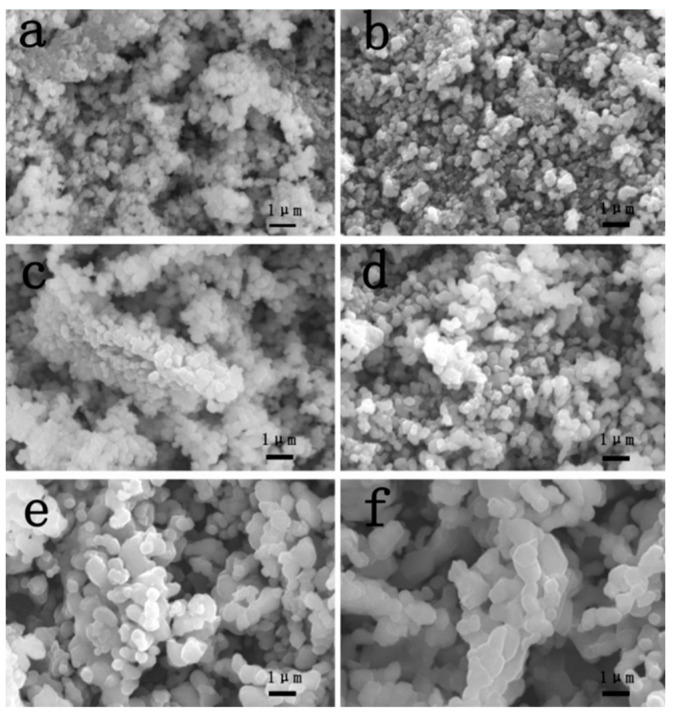
SEM images of citric acid-assisted NCM111 samples synthesized at different temperatures for 16 h. (**a**) NCM-700 °C (**b**) NCM-750 °C, (**c**) NCM-800 °C, (**d**) NCM-850 °C, (**e**) NCM-900 °C, and (**f**) NCM-950 °C, adopted from ref. [97].

In the pursuit of optimizing NCM111 cathode materials for LIBs, a comprehensive exploration of LiNi_1/3_Co_1/3_Mn_1/3_O_2_ powders has been undertaken through a citric acid-assisted sol–gel method, considering a range of temperatures (700, 750, 800, 850, 900, and 950 °C) for final calcination [97]. Using an ultrasonic treatment, metal-citrate gel precursors were prepared by dissolving a stoichiometric amount of metal acetate salts (lithium, nickel, cobalt, and manganese) and a saturated citric acid (chelating agent) in deionized water. The reaction proceeded by heating the mixture on a hot plate while stirring to form a viscous gel. The article describes the next steps involved in oven-drying the resultant gel precursor at 100 °C, pre-heating the powder at 450 °C, grinding into a fine powder with a mortar and pestle, and calcining the powder at different temperatures in a muffle furnace under an air atmosphere. Finally, after allowing the powder to cool naturally to room temperature, the final NCM111 material was obtained. The resulting morphology of the samples varied with the temperature rate, and the particles were largely nano sized with high agglomeration, as illustrated in Figure 2. At low calcination temperature, it seems that the ordered layered crystal structure was not yet fully developed. The particle distribution was inhomogeneous with an obscure grain boundary. The reasonable and uniform particle size distribution was observed only when the calcination temperature increased to 850 °C.

**Figure 3:**
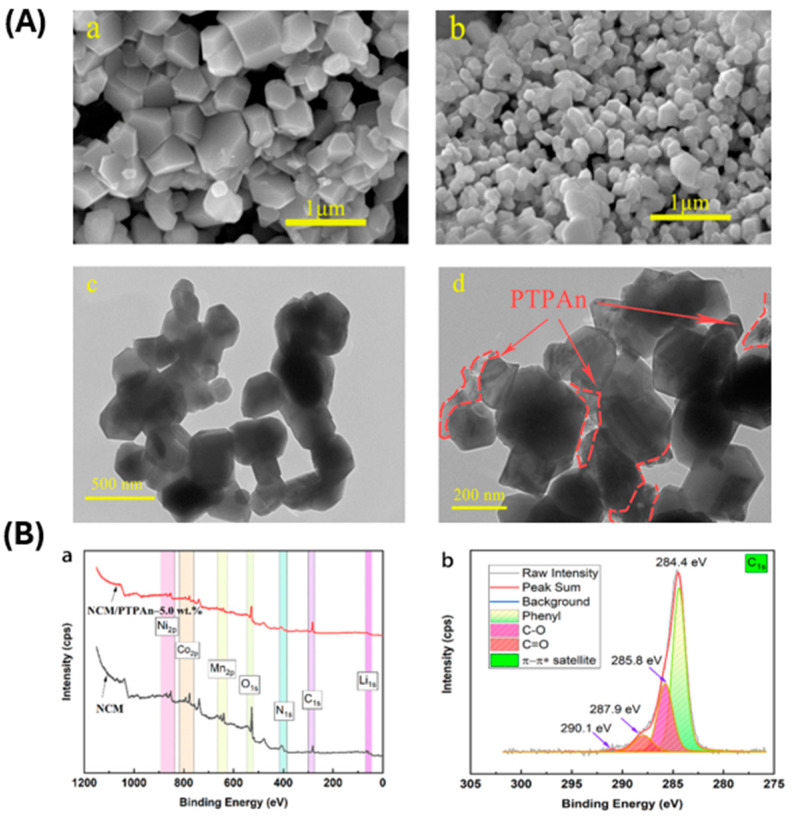
(**A**) SEM images of ethylene glycol-assisted pristine NCM111 (**a**,**c**) and polytriphenylamine (PTPAn)-coated NCM111 (**b**,**d**) samples. (**B**) The XPS study results: (**a**) full spectra, and (**b**) high-resolution spectra of C 1s; reproduced with permission from ref. [100]; copyright, Elsevier.

It is also feasible to achieve the smooth and regular particle shape of NCM111 by substituting an aqueous solvent for ethylene glycol in the sol–gel method. This results in distinct grain boundaries and homogeneous particle distribution, as illustrated in Figure 3A. Zhu et al. conducted a comprehensive investigation utilizing the ethylene glycol-assisted gel precursor for the synthesis of NCM111 [98]. The synthesis procedure was identical except for the step where the metal salts and citric acid chelating agent were mixed in ethylene glycol. With a higher c/a value and a lower degree of cationic mixing Li^+^/Ni^2+^, this NCM111 material had a more organized structure and a more regular particle shape. This structural characteristic facilitated rapid ion diffusion for Li^+^ ions by shortening their diffusion length. The as-prepared NCM111 exhibited a remarkable first discharge capacity of 208 mAh g^−1^ at the 2.5 to 4.5 V voltage window at 0.2 C, retaining 133 mAh g^−1^ after 200 cycles. In addition to this, it also demonstrated an impressive rate capacity of 143 mAh g^−1^ at 2 C current rate. These findings highlight the fact that NCM111 prepared using this method can deliver high discharge capacity, remarkable rate capability, and impressive cycle stability. A study like this is a valuable contribution to the understanding of how other layered oxides may be synthesized as potential cathode materials for lithium-ion batteries. Surface coating is considered a noteworthy exploration of strategies to improve the performance of NCM cathode materials. G. Sun et al. observed a substantial increase in discharge capacity, elevating from 87 to 138 mAh g^−1^ at 5 C, following the application of a La_2_O_3_ coating [99]. Yang et al. employed a synthetic approach to develop polytriphenylamine (PTPAn)-coated NCM111, with the composition containing 5.0 wt.% PTPAn exhibiting an impressive discharge capacity of 127 mAh g^−1^ at 5 C [100]. In this study, pristine NCM111 with a layered structure was synthesized by ethylene glycol-assisted gel precursor formation approach as mentioned above. Ultrasound-assisted homogeneous dispersion of pre-synthesized PTPAn polymer was then executed on the NCM111 powder. The surface-sensitive XPS analysis confirmed the PTPAn polymer coating (Figure 3B). These diverse methodologies underscore the versatility in tailoring the electrochemical characteristics of NCM cathode materials, providing valuable insights for advancing the performance of lithium-ion batteries.

However, despite its popularity, the NCM111-layered cathode lacks the safety and cost benefits associated with LiMn_2_O_4_ and LiFePO_4_ cathodes, which are crucial considerations for electric vehicle batteries. Cobalt is a critical resource. It has been reported that cobalt supply deficits may occur as early as 2030. Cobalt exports to the world are largely dependent on the Democratic Republic of the Congo (DRC), whose geopolitical instability and unethical working conditions may cause exports to cease. For example, a cobalt crisis occurred in 1978. Cobalt holds one-third share in the NCM111 material. Introducing a low-cobalt series can help to minimize this concern. Recently, Sourav Mallick and colleagues provided a concise overview of low-cobalt active cathode materials designed to boost the energy density of LIBs [101]. Their report delves into various synthesis methods employed for the development of these materials and explores strategies for enhancing their performance characteristics. Recognizing the strong influence of Ni-content on the specific capacity of NCM, increasing the Ni-content has proven to be an effective strategy for enhancing the charge–discharge capacity [102]. This approach has led to the development of cost-effective and high-capacity (>200 mAh g^−1^) Ni-rich NCM materials, such as LiNi_0.8_Co_0.1_Mn_0.1_O_2_, which are well suited for hybrid and electric vehicle batteries [103,104]. This can revolutionize the electric vehicle industry, leading to more efficient and long-range driving batteries. This can, in turn, make electric vehicles more affordable and more accessible, making them a viable means of transportation. Several research groups have employed gel-precursor-assisted method to synthesize a range of high-nickel NCMs, including LiNi_0.8_Co_0.1_Mn_0.1_O_2_ [105], LiNi_0.9_Co_0.05_Mn_0.025_Mg_0.025_O_2_ [106], and LiNi_0.7_Co_0.15_Mn_0.15_O_2_ [107].

In a notable study in 2013, Lu et al. [105] highlighted that LiNi_0.8_Co_0.1_Mn_0.1_O_2_ (NCM811), synthesized by citrate-based gel precursor, exhibited advantages such as lower Li^+^/Ni^2+^ cationic mixing, reduced particle aggregation, and a larger BET surface area in comparison to NCM derived from co-precipitation. According to the morphology inspection results, the primary particle size of the co-precipitation sample was smaller than that of the gel precursor-derived sample, but it contained a significant amount of aggregation. Because of the aggregates in the co-precipitation sample, it was more difficult to break them apart during electrode preparation. This resulted in a smaller total surface area, and therefore a smaller amount of active surface area in contact with the electrolyte as compared to the gel precursor-derived sample, which had a more porous structure. Their electrochemical performance was deeply influenced by these morphological characteristics. In this study, the gel precursor was formed by mixing the metal (lithium, nickel, manganese, and cobalt) nitrate salts and the citric acid (the metal ion: citric acid molar ratio was equivalent to 1:1) in distilled water. Ammonia water was added to adjust the pH to 7.0. A viscous gel subsequently obtained after the mixture was evaporated at 80 °C under vigorous stirring. To decompose the organic components of the gel, it was dried and pre-calcined in the air at 480 °C. In the final step, NCM811 samples were obtained by calcining the decomposed gel precursor at 750 °C. Another study then used a similar method to synthesize a Mg-doped high-nickel NCM, LiNi_0.9_Co_0.05_Mn_0.025_Mg_0.025_O_2_ [106]. For preparing gel precursor, a similar approach was followed. For doping purposes, only magnesium nitrate salt was included. The solvent evaporated continuously at 60 °C until it formed a viscid green xerogel. For the decomposition of the organic constituents and nitrate components, the xerogel was dried at 120 °C overnight in an oven. Then, it was crushed and heated to 550 °C in the air. Following that, the sample was re-ground, pelletized, and calcined at different temperatures. Given the opportunity to add Li salt early in the reaction, citric acid played a crucial role as the complexing agent in both cases, enabling homogeneous mixing of Li with other metal salts. The optimized calcination temperature of Mg-doped high-nickel NCM was 700 °C. The optimized sample exhibited an initial discharge capacity of 201 mAh g^−1^.

**Figure 4:**
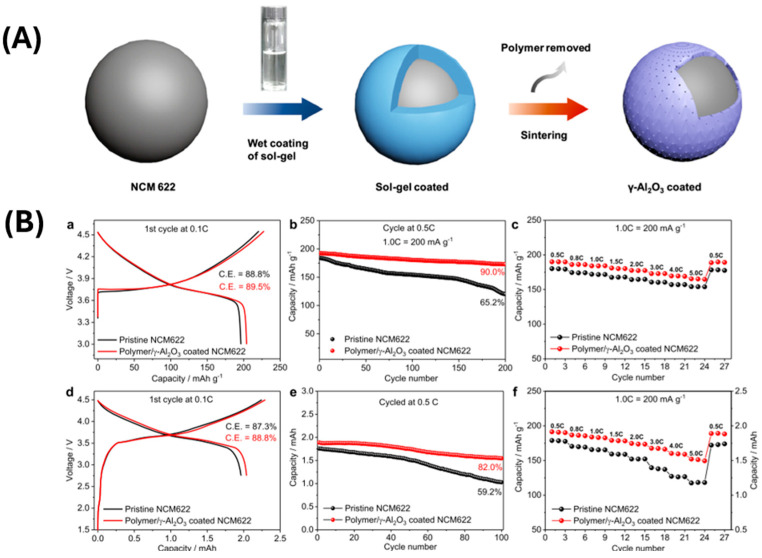
(**A**) NCM622 particle surface coating schematic illustration and (**B**) comparative high-voltage performances of coated and pristine NCM622 electrodes. Half-cell comparison of the (**a**) initial cycle capacity at 0.1 C, (**b**) cycling performance at 0.5 C, and (**c**) rate capability performance, where the charge rate was 0.5 C. (**d**–**f**) Relative comparisons for their full battery performance. 1 C = 200 mA g^−1^, adopted from ref. [108].

The sol–gel process can also be used to coat an electrode surface with a metal oxide layer. Wu et al. [108] introduced an innovative approach for enhancing the performance of a layered LiNi_0.6_Co_0.2_Mn_0.2_O_2_ (NCM622) cathode at high voltage through the application of a microporous Al_2_O_3_ sol−gel coating. A water-soluble polymeric gelator, polyvinyl alcohol (PVA), was applied with an aqueous solution of aluminum tri-sec-butoxide and HNO_3_ to produce a surface coating layer. The solution was refluxed at 90 °C to obtain a viscous gel. The NCM622 active material was mixed with this gel precursor solution in water while stirring. After the evaporation of the solvent, the vacuum-dried residue powder was calcined at 550 °C to obtain the desired coating layer. The NCM622 cathode coated with polymer/γ-Al_2_O_3_ exhibited a slightly increased capacity compared to the pristine counterpart (203.97 mAh g^−1^ vs. 196.07 mAh g^−1^). This improvement is attributed to the γ-Al_2_O_3_ coating layer, which enhanced interface stability and Coulombic efficiency (C.E., 89.8 vs. 88.8%). The chosen polymer aided in forming a flexible film, leaving micropores after sintering. The polymer/γ-Al_2_O_3_-coated NCM622 demonstrated significantly enhanced electrochemical performance when cycled at a high voltage, exhibiting 22.8% and 26% higher cycling stability and rate capabilities, respectively, compared to the pristine counterpart (Figure 4).

**Figure 5:**
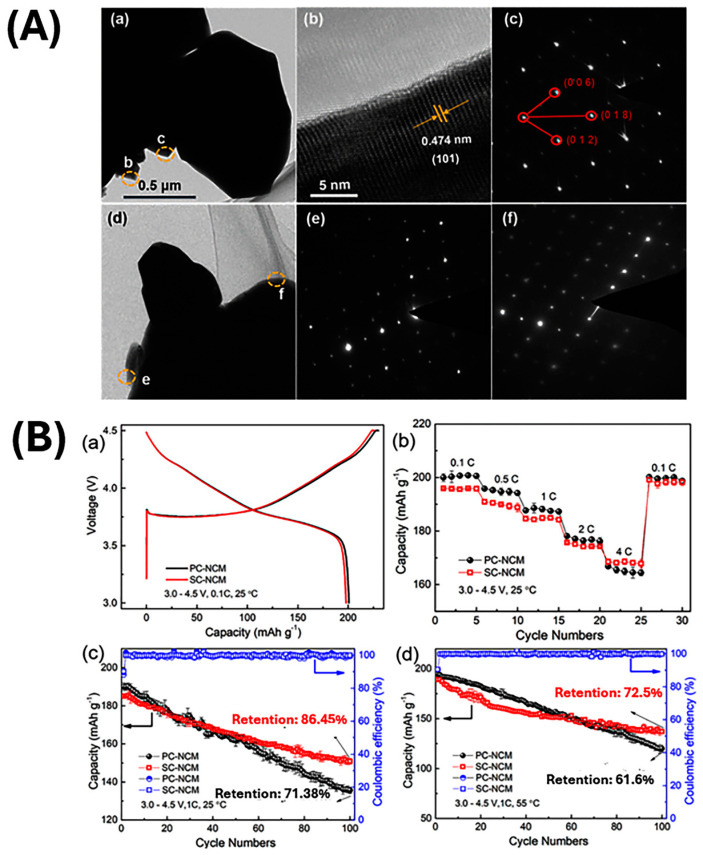
(**A**) Crystallographic orientation alignment of single crystal NCM: (**a**) TEM image, (**b**) HRTEM image of the circular area b in (**a**), (**c**) SAED pattern of the circular region c in (**a**), (**d**) TEM image of another particle, and SAED patterns of the (**e**) circular region e in (**d**), (**f**) circular region f in (**d**). (**B**) Electrochemical performance comparison between SC-NCM and PC-NCM cathode materials: (**a**) charge–discharge curves at 0.1 C-rate, (**b**) rate capabilities, and cycling performance at (**c**) room temperature and (**d**) 55 °C; reproduced with permission from ref. [107]; copyright, American Chemical Society.

A single-crystal cathode is generally more resistant to cyclic degradation and provides better safety than a polycrystalline cathode. In preparation of the NCM single-crystal cathode, several methods are used, which require accurate control of the reactant concentration and feeding rate, and subsequent lithiation is also necessary. Due to the difficult nature of the reaction condition, byproducts are sometimes inevitably formed, leading to impure phases [109]. Guo et al. [107] conducted a successful investigation through the gel precursor formation approach to obtain a single-crystal LiNi_0.7_Co_0.15_Mn_0.15_O_2_ cathode. The gel precursor formation started with the mixture of the corresponding metal acetate salts dissolving in deionized water. The aqueous solution of citric acid gelator was slowly poured into the metal cation solution in a water bath at 50 °C under stirring. The mixture pH was adjusted to 9.0 by adding ammonia–water gradually. The gel was formed by the expulsion of water. The resultant gel was pre-heated at 200 °C and then calcined at different temperatures (such as 860, 890, 920, and 940 °C) in an O_2_ atmosphere. Finally, the single-crystal LiNi_0.7_Co_0.15_Mn_0.15_O_2_ cathode was obtained after being naturally cooled. The optimized calcination temperature was 890 °C. The TEM images and corresponding SAED pattern confirmed the single-crystal NCM formation (Figure 5A). One of the greatest advantages of the gel precursor-derived synthesis route for this single-crystal material was that it did not require any crushing or the addition of any molten salt during synthesis. When comparing the electrochemical performance, the single-crystal NCM (SC-NCM) outperformed its polycrystalline NCM (PC-NCM) counterpart, demonstrating better capacity retention (86.34% vs. 71.38% at room temperature and 72.45% vs. 61.60% at 55 °C, at a 1 C rate after 100 cycles (Figure 5B). Analysis of the surface pore structure indicates that SC-NCM had superior discharge performance because to its higher specific surface area and increased ability to generate lithium-ion transmission channels. The PC-NCM, on the other hand, had a lower specific surface area but a higher pore volume. This might be because the primary particles were accumulated in nanoscale gaps, which diminished the structural stability of the material. Large pore volumes of materials may cause particle slippage and structure collapse during battery cycling.

### 3.2. Lithium Rich Nickel Cobalt Manganese Oxide (LR-NCM)

**Figure 6:**
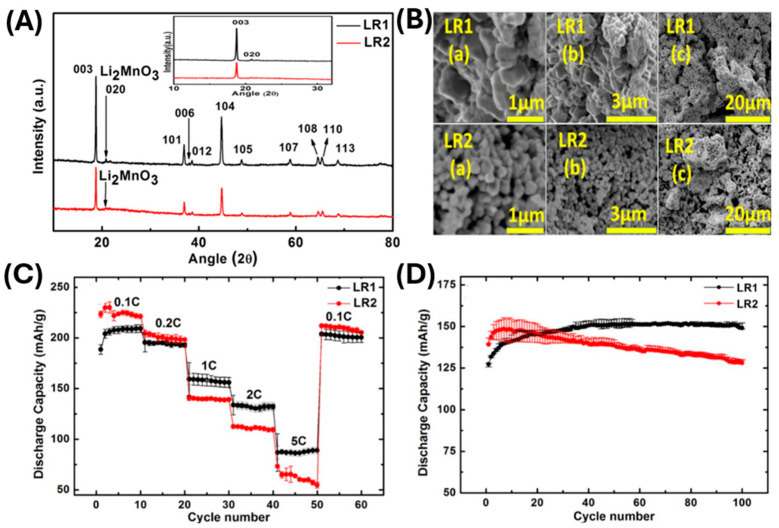
Two as-synthesized Li-rich NCM cathodes: (**A**) XRD pattern, (**B**) SEM images, (**C**) rate capabilities, and (**D**) cycling performance; adopted from ref. [110].

Two lithium-rich cathode materials, denoted as Li_1.2_Mn_0.51_Ni_0.145+x_Co_0.145−x_O_2_ (where x = 0 (LR1) or 0.0725 (LR2)), were synthesized successfully through an innovative sol–gel technique employing glycerol as both the solvent and reagent [110]. The study used cornstarch, a biomolecule, as a gelling agent by high-speed mixing with corresponding metal acetate salts dissolved in a glycerol solvent at 100 °C. The gel appeared after 3–4 h, was dried at 250 °C, and was subjected to stepwise high-temperature heat treatment in two consecutive stages (450 °C and 900 °C). The resulting materials had typical Li-rich phases and well-defined crystallinity, although particle size and morphology varied between the two samples (Figure 6A,B). SEM images showed that LR1 consisted of rectangular prism and hexagonal prism nanorod particles with agglomerates. In contrast, LR2 has semi-spherical particles. Nanorods with rectangular and hexagonal prisms and short b-axis distances were found to be favorable for Li^+^ diffusion during charge/discharge [111]. As can be seen from their superior cycling and rate capability performance (Figure 6C,D), LR1 exhibited a desirable morphology that promoted a smooth path for Li^+^ diffusion.

**Figure 7:**
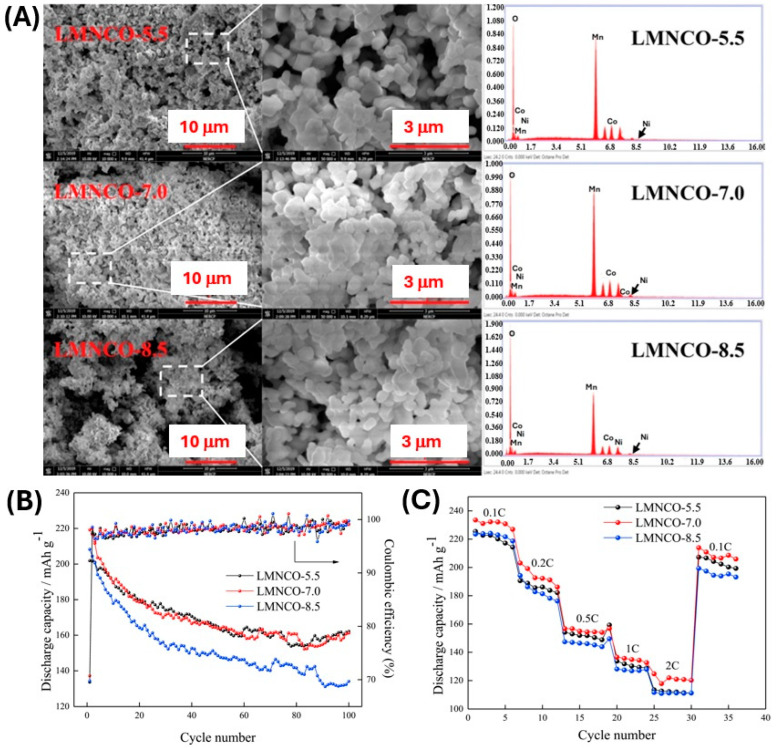
Three Li-rich NCM cathodes synthesized by pH = 5.5, 7.0, 8.5 variation: (**A**) SEM images and EDS spectra, (**B**) cycling performance at 0.2 C, and (**C**) rate capabilities; reproduced with permission from ref. [112]; copyright, Elsevier.

Using sol–gel method, Chen et al. [112] explored the synthesis of a nano-sized lithium Li_1.2_Mn_0.54_Ni_0.13_Co_0.13_O_2_ cathode materials using DL-lactic acid as a chelating agent in 2021. They produced three distinct samples, denoted as LMNCO-5.5, LMNCO-7.0, and LMNCO-8.5, by adjusting the pH = 5.5, 7.0, and 8.5, respectively, during the chelation process. All samples had well-crystallized, smooth surfaces with obvious grain boundaries, as revealed by SEM images (Figure 7). Basic (LMNCO-8.5) and neutral (LMNCO-7.0) conditions resulted in mostly quasi-spherical nanoparticles, while the acidic (LMNCO-5.5) condition showed roughly polyhedral nanoparticles. After 100 cycles, the low-pH sample (LMNCO-5.5) displayed highest specific discharge capacity (162.18 mAh g^−1^) and favorable cycling stabilities with superior capacity retention rate (80.32%). While the high-pH sample (LMNCO-8.5) showed a faster capacity decay, possibly attributed to the superior chelating effect of lactic acid on metal ions under acidic conditions. Interestingly, the neutral-pH sample (LMNCO-7.0) demonstrated superior rate performance compared to the other samples, indicating accelerated lithium-ion and electron migration rates at a pH = 7 for the LMNCO-7.0 sample.

**Figure 8:**
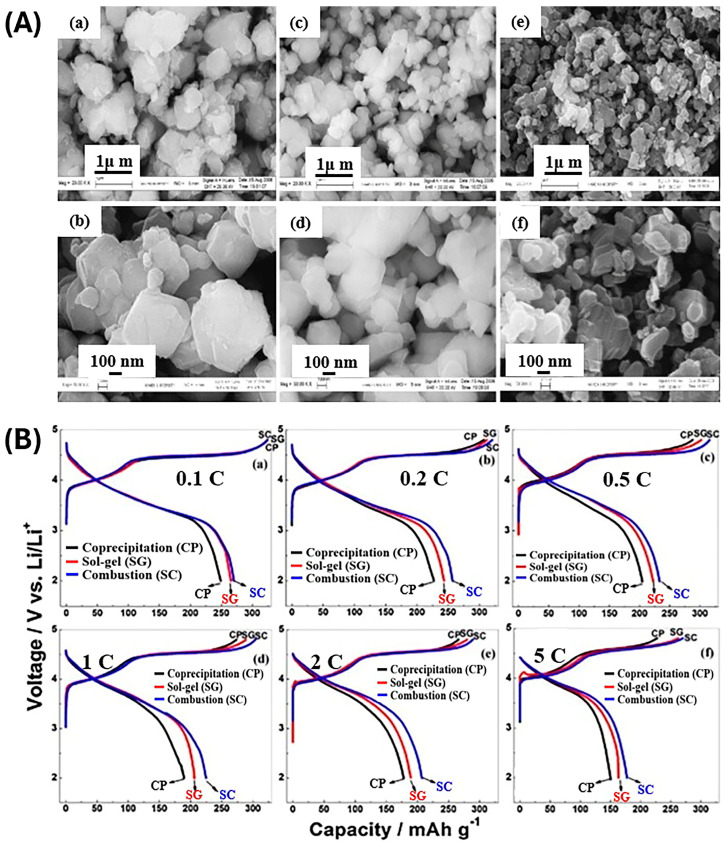
(**A**) SEM images of Li[Li_0.2_Mn_0.54_Ni_0.13_Co_0.13_]O_2_ cathode synthesized by three different methods: (**a**,**b**) co-precipitation (CP), (**c**,**d**) sol–gel (SG), (**e**,**f**) sucrose combustion (SC). (**B**) The initial charge/discharge curves of those cathodes at (**a**) 0.1 C, (**b**) 0.2 C, (**c**) 0.5 C, (**d**) 1 C, (**e**) 2 C, and (**f**) 5 C-rates; reproduced with permission from ref. [113]; copyright, Elsevier.

Zheng et al. [113] compared three synthesis methods: sol–gel, co-precipitation, and combustion method, for preparing Li[Li_0.2_Mn_0.54_Ni_0.13_Co_0.13_]O_2_ cathode. In direct comparison with the co-precipitation (CP) and combustion methods (SC), the sample produced through the sol–gel method (SG) exhibited superior cycling stability (Figure 8). In sol–gel method, citric acid as chelating agent with metal nitrate salts. Ammonium hydroxide was used to adjust the pH of the solution to 7.0–8.0. The particle size variation was consistent with the trend that CP-material was larger than SG-material and SC-material was smaller than SC-material.

Gel precursor approach is also effective to prepare doped lithium-rich NCM. In a significant contribution to the enhancement of lithium-ion battery cathode materials performance, Nisar et al. conducted a detailed investigation into the synthesis of Cr-doped lithium-rich phases, specifically Li_1.2_Ni_0.16_Mn_0.56_Co_0.08−x_Cr_x_O_2_ (where x = 0.0, 0.01, and 0.02) denoted as NCM-Cr, utilizing the sol–gel technique [114]. As far as particle morphology is concerned, all samples demonstrated a similar rock-like grain morphology. Increasing the Cr-content caused the particle size to decrease. This study involved a comprehensive analysis, employing techniques such as XRD, XPS, and elemental mapping, to confirm the homogeneous distribution of Cr in the crystal structure. The electrochemical performance of the Cr-doped materials was systematically compared with the undoped pristine sample. The results demonstrated that the Cr-doped samples exhibited significantly enhanced cycling stability, achieving 100% capacity retention after 50 cycles, compared to a mere 44% for the undoped sample. Moreover, the Cr-doped materials displayed superior electrochemical performance at higher C rates when compared to the undoped NCM. The latter experienced rapid capacity fading from 220 to 50 mAhg^−1^ at rates from 0.1 to 1 C, respectively. Notably, the Cr-containing materials exhibited minimal signs of voltage fading during cycling, attributed to the stabilizing effect of chromium on the crystal lattice. This research holds promise for advancing the design of lithium-ion battery cathodes, offering improved cycling stability and electrochemical performance.

When different chelating agents are utilized in the sol–gel synthesis, the morphology as well as the electrochemical performance can be altered. A study conducted in 2022 investigated the synthesis of Li[Li_0.2_Ni_0.3_Mn_0.7_]O_2_ cathode using the gel precursors, with a focus on the influence of three different organic acids as complexing agents [115]. The composites, labeled as LNMO-OX, LNMO-TA, and LNMO-AS, were prepared using oxalic acid, tartaric acid, and ascorbic acid, respectively. Notably, the AS-LNMO demonstrated superior capacity at various C-rates and cycling stability after 200 cycles compared to the other samples. This was due to its relatively small particle size, elevated carbon content, and an increased number of activated Li_2_MnO_3_. In contrast, OX-LNMO, which had a larger particle size, exhibited the worst capacity fading.

### 3.3. Lithium Nickel Cobalt Aluminum Oxide (NCA)

Researchers found the NCA cathode material to have high energy density and stability, making it suitable for electric vehicle batteries. Since then, its widespread adoption has made it one of the dominant cathode materials in electric vehicle industries.

Hamad et al. employed the sol–gel synthesis method to enhance the retention rate of a LiNi_0.8_Co_0.15_Al_0.05_O_2_ (NCA 80) cathode, using glycerol as the solvent instead of water [116]. In this study, metal (lithium, cobalt, nickel) acetate and aluminum nitrate salts mixture, dissolved in glycerol, was allowed to condense at 80 °C with stirring. This experiment was conducted with a metal salt: glycerol molar ratio maintained at 1:3, as well as the addition of cornstarch to the mixture as a gelling agent with a molar ratio (M^+^/cornstarch) of 4:1. After gel formation, the NCA precursor powder was dried at 400 °C. The dry powder was then pelletized and treated at 750 °C in air for 12 h in a high-temperature furnace. The utilization of glycerol as a solvent was effective in suppressing Ni^2+^ migration to the Li layer, thereby positively influence the Li^+^ diffusion during the charge/discharge processes. The obtained results revealed remarkable capacity retention rates of 87.6% at 0.3 C and 87.3% at 1 C. The rate capability of the cell was evaluated at different current densities, returning to 0.1 C for 70 cycles (50–120th cycle) with an outstanding capacity retention of 93.6%. In comparison, other methods showed relatively lower capacity retention percentages: ball-milling, 80.9%, 1 C at 100 cycles [117]; co-precipitation, 79.9%, 0.5C after 100 cycles [118]; solid-state, 77.36%, 1 C at 60 cycles [119].

**Figure 9:**
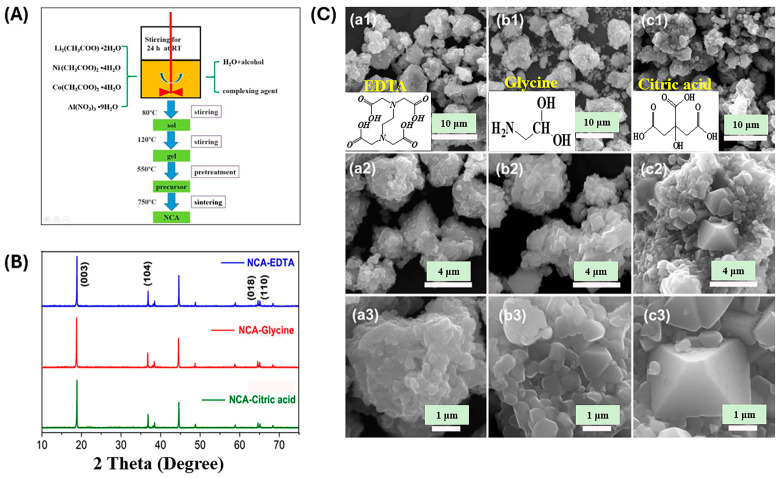
(**A**) A flow chart showing the steps involved in synthesis of the NCA cathode using the sol–gel method. Structural and morphological characteristics of three NCA cathodes prepared by different complexing agents: Citric acid, Glycine, EDTA (**B**) XRD patterns and (**C**) SEM images of NCA-EDTA (**a1**–**a3**), NCA-Glycine (**b1**–**b3**), and NCA-Citric acid (**c1**–**c3**) samples from low to high resolution; reproduced with permission from ref. [120]; copyright, Elsevier.

An insightful comparative study was conducted on the impact of complexing agents in the synthesis of NCA80 cathodes using the sol–gel method [120]. The researchers explored the use of common organic complexing agents (gelators), including EDTA, glycine, and citric acid, to prepare various NCA cathodes. The aim was to investigate the influence of complexing agents on the structural and electrochemical properties of the resulting materials. For preparing gel precursors, stoichiometric amounts of metal (lithium, cobalt, nickel) acetate and aluminum nitrate salts were dissolved in a water and ethanol mixture in an oil bath with stirring. The metal ion concentration was fixed to 1:1 with the complexing agent. Continuous stirring and solvent expulsion resulted in viscous gel. After decomposing at 550 °C, the gel was calcined at 750 °C in oxygen in a furnace, and the sample was naturally cooled to get the final product (Figure 9A). XRD spectra confirmed the successful formation of NCA cathode by all three complexing agents (Figure 9B). NCA-EDTA exhibited fine particles without nano size adherents, while the NCA-glycine demonstrated loose, fine particles with small nano particle on the particle surface. The NCA-citrate sample displayed worse morphology with the cracked small secondary particles (Figure 9C). Because of the strong complexing ability between EDTA and metal atoms, NCA-EDTA exhibited the best structural integrity when compared to other complexing agents. Notably, NCA-EDTA particles exhibited the largest c/a value and I_003_/I_104_ values. In other words, these particles demonstrated that the lattice parameters along the a-axis are the minimum, while the lattice parameters along the c-axis are the maximum. This outcome was attributed to the uniform distribution of precursor elements and particle sizes, enhancing the oxidation strength of the solid-phase reaction. The strong complexation ability of EDTA resulted in the formation of the most stable complex and relatively better electrochemical performance.

**Figure 10:**
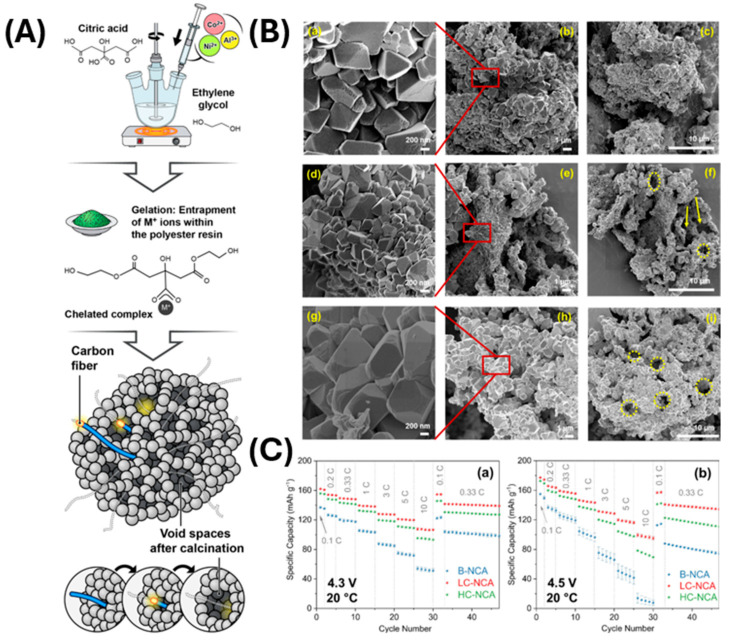
(**A**) This graphic illustrates the synthesis procedure for C-NCA materials fabricated using CF templates. (**B**) SEM images of powder samples (**a**–**c**) bare NCA (B-NCA), (**d**–**f**) CF template-assisted NCA with lower CF content (LC-NCA), and (**g**–**i**) CF template-assisted NCA with higher CF content (HC-NCA). Arrows and areas circled in yellow indicate where CF fibers existed prior to calcination. (**C**) Comparison of the C-rate performance of B-NCA (blue), LC-NCA (red), HC-NCA (green) materials at cut off voltages of (**a**) 4.3–2.9 and (**b**) 4.5–2.8 V. Reproduced with permission from ref. [121]; copyright, American Chemical Society.

Kopuklu et al. [121] conducted an in-depth investigation into the sacrificial carbon fiber (CF) template-assisted synthesis of LiNi_0.8_Co_0.15_Al_0.05_O_2_ (C-NCA) using the Pechini method—a modified sol–gel method (Figure 10A). Initially, the complete dissolution of citric acid monohydrate (the chelating agent) in ethylene glycol (EG) with a molar ratio of 1:4 was ensured by heating it at 80 °C with stirring in an oil bath under N_2_ gas purging. The purpose of this step was to minimize the entrapment of humidity from the air that might interfere with the gelation process. Using probe sonication, O_2_ plasma-treated carbon nanofibers (CFs) were dispersed in 2-isopropyl alcohol. Stoichiometric amounts of all metal chloride salts were dissolved in the CFs/alcohol dispersion. Dropwise injection of the metal–CFs solution into the citric acid solution was performed under stirring at 80 °C, followed by a small volume of ammonium hydroxide to facilitate chelation and create a gel containing the metallic chelate precursor. The gels were dried by a progressive increase in temperature from 70 °C to 120 °C for couple of days in a vacuum oven to initiate the chelation reaction and form the polymeric resin. Completely dried gels appeared to be dark, green-colored solids. They were ground into powder by an agate mortar and pre-calcined at 500 °C under pure oxygen to produce NCA-gel precursor (Ni_0.8_Co_0.15_Al_0.05_O_2_). This oxide powder was heated with LiOH·H_2_O to a high temperature of 800 °C in a tube furnace under a pure O_2_ environment for the lithiation reaction. The particles formed around the CFs exhibited a denser packing compared to the reference bare NCA (B-NCA), which was synthesized in the absence of the CF template. This assembly of particles around the CFs with anisotropic surfaces and tetra-decahedron-type morphology was found to facilitate ion transport and stabilize the structure, particularly beneficial for high-voltage and -temperature operations (Figure 10B). The C-NCA demonstrated a notable reversible capacity of 106 mAh g^−1^ at a high C-rate of 10 C and impressively retained 96% of its initial capacity as the C-rate was reverted to 0.1 C at cut-off voltages of 4.3–2.9 and 4.5–2.8 V (Figure 10C). The state-of-health (SOH) of C-NCA full-cells remained at 70% even after 200 cycles at 0.33 C within the voltage range of 2.8–4.3 V. This innovative approach highlights the potential of sacrificial CF templates in enhancing the electrochemical performance and longevity of Li-ion battery cathodes.

The sol–gel process is also capable of coating the NCA electrode surface with a protective metal oxide layer. He et al., in 2019, introduced a novel approach by demonstrating the exceptional cycling and rate capacity retention of NCA80 cathode material through the incorporation of a Li_2_TiO_3_ nanoparticles (NLTO-NCA) layer [122]. Briefly, lithium nitrate salt and titanium butoxide were mixed in water with the chelating agent, CH_3_COOH, with stirring. Later, PEG600 was added at 3 wt.% to the mixture to obtain a sol. A water bath at 60 °C was then used to heat the sol until it was completely transformed into a gel. As soon as the gel had been obtained, it was dried at 120 °C and calcined at 700 °C for 4 h in air to yield a powder. Following cleaning with ionized water several times, the powder was dried at 80 °C overnight to prepare nano-Li_2_TiO_3_. The particles were sonicated in isopropanol to obtain a suspension. The pristine NCA material was added to the suspension and magnetically stirred in an oil bath at 95 °C. After a while, the isopropanol volatilized, leaving a powder. For the preparation of Nano-Li_2_TiO_3_-coated NCA, this powder was calcined at 600 °C in flowing oxygen gas. The coated material maintained the original LiNi_0.8_Co_0.15_Al_0.05_O_2_ bulk structure while exhibiting a significantly reduced lithium residue on the surface. This coating, through a sol–gel process, resulted in a remarkable improvement in electrochemical performance, particularly enhancing the cycling stability at an elevated temperature of 60 °C. After 200 cycles at a rate of 1 C, the NLTO-NCA coating demonstrated an impressive capacity retention of 91.32%. The key factor contributing to the enhanced cycling performance is attributed to the innovative sol–gel synthesized Li_2_TiO_3_ nanoparticle-coating method. This method effectively avoids Ti^4+^ doping, preventing the aggravation of Li^+^/Ni^2+^ disorder and achieving superior electrochemical performance for the coated material. Table 1 summarizes the electrochemical performance of all reported sol–gel-assisted layered cathode materials.

### 3.4. Lithium Iron Phosphate (LiFePO_4_)

In 1997, Goodenough and his colleagues [123] suggested olivine-type lithium iron phosphate (LiFePO_4_) as a highly promising cathode material. This new class of cathodes offers several benefits, such as a high specific capacity (170 mAh g^−1^), improved coulombic efficiency, a consistent charge–discharge potential, cost-effectiveness, and eco-friendliness [124]. The sol–gel method was reported to fabricate LiFePO_4_ using an aqueous solution of citric acid for chelation and gelation [125]. The sol solution was formed by mixing iron (Fe) powder, citric acid, phosphoric acid (H_3_PO_4_), lithium hydroxide monohydrate (LiOH·H_2_O), and sucrose (carbon source) in water by continuous stirring. To obtain a gel, the solution was evaporated at 60 °C for certain periods, and then the gel was oven-dried at 120 °C. It is important to note, however, that the two main drawbacks of olivine-based electrodes are their low electrical conductivity (10^−9^–10^−10^ Scm^−1^) and insufficient Li-ion diffusion, which undermine the electrochemical performance of LiFePO_4_ in secondary lithium-ion batteries [126].

Carbon-coated LiFePO_4_ cathode materials were successfully synthesized by the sol–gel method [127]. The gel precursor for this material was prepared by mixing stoichiometric amounts of NH_4_H_2_PO_4_, Fe(NO_3_)_3_·9H_2_O, and LiNO_3_ in ethylene glycol (chelating agent). Additionally, glucose, citric acid, and PEG-4000 were introduced as the carbon source, gelator, and dispersant, respectively. Upon dissolving all these raw materials and additives in distilled water, the mixture was heated in a water bath at 80 °C under stirring until a gel was formed; the dry precursors were then prepared. Prior to heating the precursors to a particular temperature (500, 600, 700, 800, and 900 °C) under a N_2_ gas flow, the precursors were decomposed at 400 °C.

A simple sol–gel method was applied by Y. Zhang et al. to prepare LiFePO_4_/C composite [128]. Citric acid was applied as the chelating agent for the metal–citrate gel precursor formation. The stoichiometric amounts of iron oxalate, ammonium dihydrogen phosphate, and lithium hydroxide were dissolved in deionized water. Citric acid and sucrose as a carbon source were added by sonication and stirring, then heated at 80 °C for condensation. After expulsion of water, the resultant viscous gel was heated at 200 °C under inert gas. A reductive atmosphere composed of 5 vol% H_2_ and 95 vol% Ar was then applied to the final step calcination at 750 °C. For comparison, LiFePO_4_ was also prepared using the same raw materials via the solid-state technique. The sol–gel synthesized LiFePO_4_/C composites exhibited a high reversible capacity of 163.5 mAh g^−1^ at 0.1 C with excellent rate capability and cycling capability. Li et al. prepared LiFePO_4_/C nanocomposites with impressive capacity and cycle performance [129]. They used FeCl_2_·4H_2_O, H_3_PO_4_, and Li_2_CO_3_ as the starting materials and citric acid as the chelating agent. The sol was prepared in anhydrous ethanol instead of water. After condensation, the resultant gel was transferred to a 60 °C blast oven for 12 h to dry it sufficiently. After pre-calcination at 350 °C in a tubular furnace under a protective N_2_ atmosphere, the temperature was raised to 650~750 °C in the same environment. Arbizzani et al. synthesized a three-dimensional carbon-coated LiFePO_4_ through sol–gel processing [130]. As an iron source and chelating agent, they used home-made iron citrate as raw material. Using a stoichiometric ratio of Fe(III)-citrate and H_3_PO_4_ and Li_3_PO_4_, the aqueous solution was heated at 60 °C with stirring to produce a concentrated solution, which was then applied to CP (carbon paper) disks to imbibe the inner fibers under vacuum. After two days of incubation at 60 °C for the formation of xerogel on the carbon fibers, the impregnated disks were heated in a tubular furnace to 700 °C under 5% H_2_-Ar gas flow.

**Figure 11:**
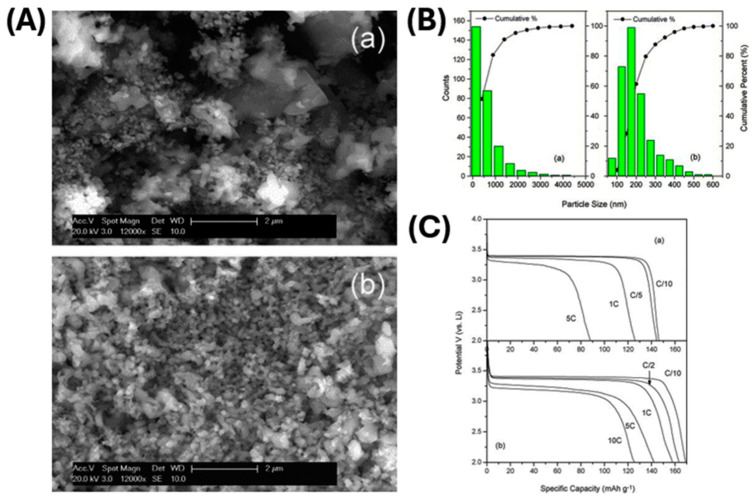
(**A**) The SEM micrographs of LiFePO_4_ powders synthesized (**a**) without and (**b**) with lauric acid. (**B**) Particle size distribution of LiFePO_4_ powders synthesized (**a**) without and (**b**) with lauric acid. (**C**) The discharge capacity profile at different C rates for LiFePO_4_ synthesized (**a**) without and (**b**) with lauric acid. Reproduced with permission from ref. [132]; copyright, Elsevier.

Chen et al. [131] used yeast as a biocarbon source with citric acid as a chelating agent in a sol–gel processed LiFePO_4_/C composite synthesis. The sol was prepared from FeCl_2_·4H_2_O, LiOH·H_2_O, and NH_4_H_2_PO_4_ raw materials. The sol was heated at 80 °C for several hours under vigorous stirring until the gel had been formed. After drying the gel, it was calcined at 700 °C, 750 °C, 800 °C, or 850 °C under a nitrogen atmosphere in a muffle furnace. Researchers also reported using lauric acid surfactant as a chelating agent to synthesize LiFePO_4_ using the sol–gel method [132]. The raw materials CH_3_CO_2_Li·2H_2_O, FeCl_2_·4H_2_O, and P_2_O_5_ were dissolved in ethanol by adjusting equal molar ratio of lauric acid with stirring. As a precaution against Fe^3+^ impurities forming, ethanol solvent was evaporated under continuous flow of ultra-high purity (UHP)-Ar, and the resultant gel precursor was calcined under H_2_/Ar = 10:90% atmosphere at 500 °C. When LiFePO_4_ was synthesized without a surfactant, it exhibited particles ranging in size from few thousand nanometers to several microns, whereas when it was synthesized with a surfactant, it exhibited uniform nanometer-sized (∼170 nm) particles that formed a porous network (Figure 11A). In particle size distribution analysis, the surfactant-treated LiFePO_4_ had an average particle size of ≈170 nm in comparison with ≈600 nm for the surfactant-free LiFePO_4_ (Figure 11B). In comparison with LiFePO_4_ synthesized without the surfactant, LiFePO_4_ synthesized with the surfactant demonstrated significantly better electrochemical properties with a specific capacity of 170 mAh g^−1^ at C/10 rate. The large particle size was the main cause for the LiFePO_4_ yield of only 146 mAh g^−1^ at C/10 rate when synthesized without the surfactant (the core of the particles remained unused) (Figure 11C).

**Figure 12:**
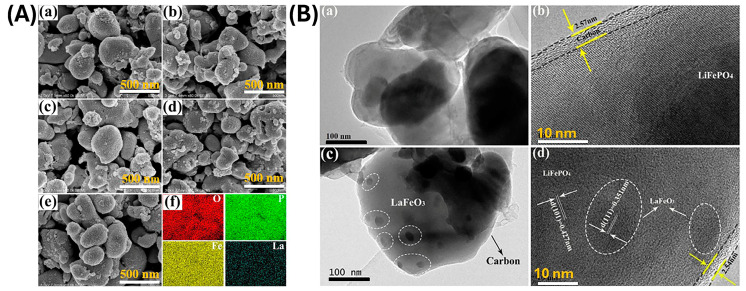
(**A**) SEM images of (**a**) LFP@C, (**b**) LFP@C-1 wt% LaFeO_3_, (**c**) LFP@C-2 wt% LaFeO_3_, (**d**) LFP@C-3 wt% LaFeO_3_, (**e**) LFP@C-4 wt% LaFeO_3_, and (**f**) element mapping of LFP@C-1 wt% LaFeO_3_. (**B**) TEM images of (**a**,**b**) LFP@C and (**c**,**d**) LFP@C-1 wt% LaFeO_3_. Herein, circles in figure **B**(**d**) show the equivalent lattice fringe (d_111_ = 0.351 nm) of LaFeO_3_. Reproduced with permission from ref. [134]; copyright, American Chemical Society.

**Figure 13:**
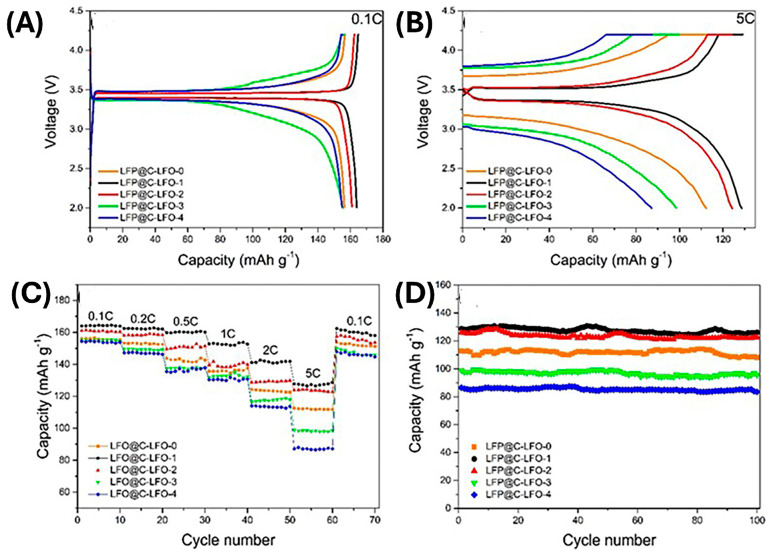
Electrochemical properties of LFP@C-LiFeO_3_-x: (**A**) first charge/discharge profiles at 0.1 C, (**B**) first charge/discharge profiles at 5 C, (**C**) rate capability, and (**D**) cycling performance at 5 C. Reproduced with permission from ref. [134]; copyright, American Chemical Society.

Sol–gel methods have been used to synthesize Mg- and Ti-doped LiFePO_4_, during which a variety of conditions of processing was applied [133]. In this study, glycolic acid was used as a chelating agent with the starting materials of iron nitrate, lithium acetate, and phosphoric acid. A solution of phosphoric acid and de-ionized water was used to dissolve the metal compounds. Glycolic acid was added to this homogeneous solution while stirring until there was a 2:1 molar ratio between glycolic acid and metal ions. The pH of the solution was adjusted to 8.5–9.5 by adding ammonium hydroxide. The condensation and gel formation processes were carried out by heating the resultant sol solution at 70–80 °C under a nitrogen atmosphere to prevent the oxidation of Fe^2+^. The dried gel precursor was subjected to pre-calcination at 500 °C before final firing at 600 °C or 700 °C for various lengths of time under an inert atmosphere. For doped samples, appropriate molar fraction of the lithium acetate was replaced by magnesium nitrate salt (in Li_0.98_Mg_0.01_FePO_4_) or titanium ethoxide (in Li_0.96_Ti_0.01_FePO_4_). The carbon coating of LiFePO_4_ was achieved using various organic additives, which were added during the intermediate grinding step before the final firing. In addition, the sol–gel method was also used to coat the LiFePO_4_ (LFP) surface by metal oxide. LaFeO_3_-coated LiFePO_4_@C cathode materials have been reported by Cui et al. using a sol–gel coating approach [134]. The first step involved dissolving analytically pure-grade lanthanum nitrate and iron nitrate salts into deionized water in a 1:1 ratio to prepare a gel precursor solution of LaFeO_3_. In the next step, the powdered LiFePO_4_@C was mixed with the homogeneous LaFeO_3_ solution at a stirring speed of 800 rpm to disperse it. Gel was formed after vacuum drying at 120 °C, which was then heat-treated at 600 °C. The mass ratio (x) of LaFeO_3_ to LiFePO_4_ was controlled by adjusting precursor solution concentration (x = 0, 1, 2, 3, and 4 wt% LFO). As far as the morphology of the samples is concerned, all samples were made up of irregular microspheres with sizes between 100 and 500 nm, composed of nanoparticles smaller than 30 nm (Figure 12A). The TEM image shows that many relatively dark spots of LaFeO_3_ grew on the surface of the synthesized LFP@C-LFO-1, while a carbon-coating layer existed in coated and uncoated LFP (Figure 12B). Increasing the amount of LaFeO_3_ tended to decrease the discharge specific capacity value of LFP@C-LFO because of its inert nature. LFP@C-LFO-1 with 1 wt% LaFeO_3_ coating had the best rate capability (Figure 13). Table 2 summarizes the electrochemical performance of some reported sol–gel-assisted olivine LiFePO_4_ cathode materials.

## 4. Conclusions and Future Direction

In conclusion, lithium-ion batteries can benefit significantly from sol–gel methods for improving their electrode electrochemical properties. Applying this synthesis approach significantly improves the reversible capacity and the cycling behavior of the NCM cathode. Additionally, sol–gel coatings lend NCM cathodes greater tolerance for overcharging. A higher c/a value and a lower degree of cationic mixing Li^+^/Ni^2+^ in NCM synthesized by gel precursor was reported. Single-crystal NCM synthesis is also feasible from a gel precursor. This development opens up new possibilities for high-performance batteries in electric vehicles and portable electronics. Single-crystal NCM can enhance the energy density and longevity of these batteries, addressing some of the primary concerns in modern battery technology. In the case of lithium-rich NCM, gel precursor formation is an easy and effective way to obtain layered compounds with high phase purity. By controlling the pH of the solution during gel formation and doping with heteroatoms, it is possible to increase the structural stability of the cathode material during cycling. In direct comparison with the co-precipitation (CP) and combustion methods (SC), the sample produced through the sol–gel method (SG) exhibited superior cycling.

With nickel-rich NCA, the sol–gel process greatly reduces the extent of disordering of the cation distribution in the crystal lattice due to effective mixing. In contrast, traditional solid-state synthesis methods often suffer from poor mixing, leading to significant cation disorder. Additionally, these methods usually require higher temperatures and longer processing times. The sol–gel process, therefore, not only improves homogeneity but also enhances overall material performance. The utilization of a carbon fiber template in the synthesis of NCA80 offers a means to customize the primary particle morphology through a straightforward sol–gel method. This approach not only enhances the transportation of Li^+^ ions but also simplifies the incorporation of aluminum into the LiMO_2_ structure. Consequently, it results in an improved structural and thermal stability of the nickel-rich positive electrodes for lithium-ion battery (LIB) cells. Notably, the electrochemical behavior exhibits intriguing variations with varying amounts of precursors. As compared with bare-NCA and high carbon fiber template-assisted HC-NCA, low carbon fiber template-assisted LC-NCA demonstrated the most favorable rate performance and diffusion coefficient, highlighting its superior electrochemical characteristics. Among the common chelating agents, EDTA showed the strong complexation ability and resulted in the relatively better electrochemical performance. The sol–gel process is also capable of coating the NCA electrode surface with a protective Li_2_TiO_3_ nanoparticle layer. This enhances its reversibility, cycling behavior, and overcharge ability, making it a promising industrial candidate. Various composites based on LiFePO_4_ can be prepared by using gel precursor, and their capacity, rate capability and cycling stability are greatly improved by doping and other modification of sol–gel methods. The gel precursor was proved successful for effective coating of carbon and/or metal oxide on LiFePO_4_. After gel precursor-assisted LaFeO_3_ coating, the rate capability was greatly improved.

The use of gel precursors may result in the introduction of new and better cathodes in the future, which will possess a variety of unique properties. One of the greatest advantages of starting a synthesis in a solution state is that homogeneity can be maintained, which is of great importance when generating phase-pure solids. Another main advantage of the sol–gel method is the possibility to prepare nanomaterials. To date, the preparation of commercially popular LIBs cathode materials from gel precursors widely has used small molecule gelators like citric acid, EDTA, glycine, etc., and polymeric gelators like ethylene glycol. Moreover, biomolecules, specifically corn starch, are proved effective for LIBs cathode synthesis. Despite this, many synthetic polymers, such as PVA, PEG, and PVP, as well as biopolymers, such as dextran, gelatin, chitosan, and alginate, are still unexplored for preparing the LIB cathode materials required for commercial battery applications. In the future, there is scope to use a large number of organic acids, biomolecules, and synthetic/natural polymers in the sol–gel method to fabricate cathode materials with tuned morphology and improved electrochemical performance.

## Figures and Tables

**Table 1:** Electrochemical performance of sol–gel synthesized layered cathode materials reported in the literature.

Cathode	Specific Capacity/Current Rate	Capacity Retention	Rate (mAh g^−1^)	References
LiNi_1/3_Co_1/3_Mn_1/3_O_2_	199.9 mAh g^−1^/0.1 C	71.8% @ 80 cycles	------	[97]
LiNi_1/3_Co_1/3_Mn_1/3_O_2_	208.0 mAh g^−1^/0.2 C	66.3% @ 100 cycles	83.0@5C	[98]
LiNi_1/3_Co_1/3_Mn_1/3_O_2_/PTPAn	223.7 mAh g^−1^/0.2 C	84.3% @ 100 cycles	127.3@5C	[100]
LiNi_0.8_Co_0.1_Mn_0.1_O_2_	200.0 mAh g^−1^/0.5 C	75.8% @ 100 cycles	140.0@5C	[105]
LiNi_0.9_Co_0.05_Mn_0.025_Mg_0.025_O_2_	201.0 mAh g^−1^/0.1 C	99.3% @ 10 cycles	168.0@2C	[106]
SC-LiNi_0.7_Co_0.15_Mn_0.15_O_2_	184.6 mAh g^−1^/0.1 C	86.3% @ 100 cycles	171.0@4C	[107]
LiNi_0.6_Co_0.2_Mn_0.2_O_2_/Al_2_O_3_	203.9 mAh g^−1^/0.1 C	90.0% @ 200 cycles	177.5@5C	[108]
Li_1.2_Mn_0.51_Ni_0.145+x_Co_0.145-x_O_2_	223.0 mAh g^−1^/0.1 C	98.0% @ 100 cycles	89.0@5C	[110]
Li_1.2_Mn_0.54_Ni_0.13_Co_0.13_O_2_	232.3 mAh g^−1^/0.1 C	83.9% @ 50 cycles	120.0@2C	[112]
Li[Li_0.2_Mn_0.54_Ni_0.13_Co_0.13_]O_2_	265.0 mAh g^−1^/0.1 C	83.8% @ 30 cycles	164.0@5C	[113]
Li_1.2_Ni_0.16_Mn_0.56_Co_0.08−x_Cr_x_O_2_	200.0 mAh g^−1^/0.1 C	100.0% @ 50 cycles	150.0@1C	[114]
Li[Li_0.2_Ni_0.3_Mn_0.7_]O_2_	308.0 mAh g^−1^/0.1 C	99.3% @ 200 cycles	212.0@2C	[115]
LiNi_0.8_Co_0.15_Al_0.05_O_2_	174.8 mAh g^−1^/0.1 C	93.6% @ 70 cycles	105.0@5C	[116]
LiNi_0.8_Co_0.15_Al_0.05_O_2_	175.1 mAh g^−1^/0.2 C	97.3% @ 30 cycles	159.8@1C	[120]
LiNi_0.8_Co_0.15_Al_0.05_O_2_	169.0 mAh g^−1^/0.33 C	60.3% @ 150 cycles	120.0@10C	[121]
LiNi_0.8_Co_0.15_Al_0.05_O_2_/Li_2_TiO_3_	191.0 mAh g^−1^/0.1 C	96.6% @ 100 cycles	141.0@5C	[122]

**Table 2:** Electrochemical performance of Sol–gel synthesized olivine-type LiFePO_4_ cathode materials reported in the literature.

Cathode	Specific Capacity/Current Rate	Capacity Retention	Rate (mAh g^−1^)	References
LiFePO_4_	80.0 mAh g^−1^/0.1 C	-------	------	[125]
LiFePO_4_/C	150.0 mAh g^−1^/0.1 C	-------	------	[127]
LiFePO_4_/C	163.5 mAh g^−1^/0.1 C	91.1% @ 110 cycles	88.4@2C	[128]
LiFePO_4_/C	156.5 mAh g^−1^/0.2 C	96.9% @ 250 cycles	70.9@10C	[129]
LiFePO_4_/3D-C	100.0 mAh g^−1^/1.0 C	99.0% @ 400 cycles	85.0@5C	[130]
LiFePO_4_/C	158.3 mAh g^−1^/0.1 C	96.6% @ 100 cycles	142.0@5C	[131]
LiFePO_4_	170.0 mAh g^−1^/0.1 C	100.0% @ 30 cycles	90.0@5C	[132]
LiFePO_4_/C-LaFeO_3_	164.2 mAh g^−1^/0.1 C	97.9% @ 100 cycles	127.3@5C	[134]
LiFePO_4_/C	162.0 mAh g^−1^/0.1 C	99.1% @ 40 cycles	126.0@1.7C	[135]

## Data Availability

All data and materials are available on request from the corresponding author.
